# Anaplastic lymphoma kinase-positive large B-cell lymphoma: Clinico-pathological study of 17 cases with review of literature

**DOI:** 10.1371/journal.pone.0178416

**Published:** 2017-06-30

**Authors:** Xiang-Nan Jiang, Bao-Hua Yu, Wei-Ge Wang, Xiao-Yan Zhou, Xiao-Qiu Li

**Affiliations:** 1 Department of Pathology, Fudan University Shanghai Cancer Center, Shanghai, China; 2 Department of Oncology, Shanghai Medical College, Fudan University, Shanghai, China; Seconda Universita degli Studi di Napoli, ITALY

## Abstract

We retrospectively analysed 17 cases of anaplastic lymphoma kinase-positive large B-cell lymphoma (ALK+, LBCL) according to the morphological, immunohistochemical, molecular and clinical features, using which we intend to elucidate the clinicopathological characteristics of this rare entity. In this study, all cases de facto share common features that defined them as a single entity, and various characteristics may expand the spectrum. Among 15 cases, 60% followed an aggressive clinical course with advanced stage and high IPI scores; the median survival of these patients was only 8 months. An analysis showed that both the IPI score and the Ann Arbor stage were significant prognostic factors. Most patients received a chemotherapy regimen including CHOP, CHOEP, EPOCH, and CVAD, and some also underwent localized radiotherapy. However, ALK+, LBCL cases display a dismal clinical outcome and can only be cured with conventional chemotherapy protocols at the stage of localized disease. Novel front-line intensive chemotherapy regimens should therefore be evaluated in this group of patients.

## Introduction

Anaplastic lymphoma kinase-positive large B-cell lymphoma (ALK+, LBCL) was originally recognized in 1997 by Delsol and colleagues [1] and was listed as a distinct entity in the updated WHO Classification of Haematopoietic and Lymphoid Tissues [2]. It appears to be very rare because it represents less than 1% of all diffuse large B cell lymphomas (DLBCLs), which is partially due to the under-recognition of this disease [2].

Although it is rare, ALK+, LBCL has distinct characteristics and clinicopathological significance. Pathologically, ALK+, LBCL is composed of monomorphic immunoblast-like cells with round pale nuclei that contain large central nucleoli and abundant cytoplasm; ALK+, LBCL also exhibits a sinusoidal growth pattern. Immunohistochemically, the tumour cells co-express ALK (the staining pattern changes according to the gene fusion type and often shows a restricted cytoplasmic staining pattern), a panel of plasma cell markers, CD45, epithelial membrane antigen (EMA) and often contain single light-chain cytoplasmic immunoglobulin A (IgA). However, tumour cells do not express mature B-cell markers such as CD20. Cytogenetically, chromosomal translocations or rearrangements that involve the ALK locus are regarded as the hallmark of ALK+, LBCL. The most common gene rearrangement is between clathrin (CLTC) and ALK (t (2; 17) (p23; q23)), which results in the CLTC-ALK chimeric protein, although other types of fusions have also been described, such as the SQSTM1-ALK variant translocation [3]. Clinically, ALK+, LBCL is a more aggressive disease than typical DLBCL. In terms of treatment, response to conventional chemotherapy is poor [4]. Due to the limited number of reported cases [5–14], a lack of awareness of this entity, and significant morphologic and immunophenotypic similarities to other haematopoietic and nonhaematopoietic neoplasms, the diagnosis of ALK+, LBCL may be challenging. However, increased awareness of its occurrence and familiarity with its characteristic features are significant for both clinicians and pathologists, particularly with the advancements in emerging therapeutic options [15]. Herein, we present a clinicopathological analysis of 17 cases that delineate the features of ALK+, LBCL at our institution and a review of the literature to foster the idea that this tumour is an individual disease, with the hope that the morphological spectrum will be broadened and that the clinical data will be consummated.

## Materials and methods

### Patient samples

In all, 17 patients who presented with ALK+, LBCL from 2007 to 2012 were available for study from the surgical pathology and consultation files of the Department of Pathology, Fudan University Shanghai Cancer Center. All cases were histologically and immunohistochemically reviewed by two senior pathologists according to the updated World Health Organization Classification of Tumors of Haematopoietic and Lymphoid Tissues [2] to confirm the diagnosis. Available clinical data including presentations, therapy and follow-up information were evaluated and updated. The stage of disease was determined using the Ann Arbor staging system. Approval for these studies was obtained from the Institutional Review Board.

### Histology and immunohistochemistry

All specimens were formalin-fixed, paraffin embedded tissues that underwent routine haematoxylin-eosin (H&E) staining and microscopic observation. The immunohistochemical study was performed on paraffin sections according to the standard EnVision technique using a panel of monoclonal and polyclonal antibodies including those against CD10 (56C6; DAKO; dilution 1:40), Bcl6 (PG-B6P; DAKO; dilution 1:10), MUM1 (MUM1P; DAKO; dilution 1:50), Bcl2 (124; DAKO; dilution 1:50), CD20 (L26; DAKO; 1:50), CD3 (2Gv6; DAKO; dilution 1:80), CD138 (MI15; DAKO; dilution 1:50), ALK (ALK1; DAKO; dilution 1:80), and EMA (E29; DAKO; 1:40), C-MYC (Y69; Epitomics; dilution 1:50). For each antibody, appropriate positive and negative control samples were included. The immunohistochemistry results were reviewed by two independent certified pathologists.

### Cytogenetic analysis

#### Fluorescence in situ hybridization (FISH)

FISH analyses were performed on paraffin-embedded tissue sections with probes specific for *ALK* (LSI ALK, Vysis/Abbott, Downer Grove, USA) loci. The probe flanking the *ALK* gene breakpoint at 2p23.3 showed a red signal and a green signal under each corresponding laser. Interpretation of the results was based on the literature [16].

#### Reverse transcription-polymerase chain reaction (RT-PCR)

RT-PCR was performed to amplify the gene rearrangement products of immunoglobulin heavy chain (IGH) and T-cell receptor (TCR). Total RNA was extracted from formalin-fixed, paraffin-embedded sections according to the manufacturer's instructions. Synthesis of the first complementary DNA (cDNA) strand was performed by MMLV reverse transcriptase, which was followed by polymerase chain reaction (PCR) amplification. The PCR primers were designed according to the common *ALK* fusion gene type [S1 Table]. DNA sequencing was also used to confirm the *ALK* fusion gene type.

### Statistical analysis

Survival was determined from the time of diagnosis until the time of death or last follow-up. Survival curves were constructed according to the Kaplan-Meier method. Survival distributions were compared with the log-rank test. All statistical analyses were performed using STATA, version 11.0 (Stata Corporation, College Station, TX). The column graph was constructed with Graphpad prism, version 5.0. Fisher’s exact test was also performed. All *p*-values were two sided, and a *p*-value ≤0.05 was considered statistically significant. Protein expression levels were judged by H-score (positive staining intensity by positive percentage) standard.

## Results

### Clinical features

**Fig 1:**
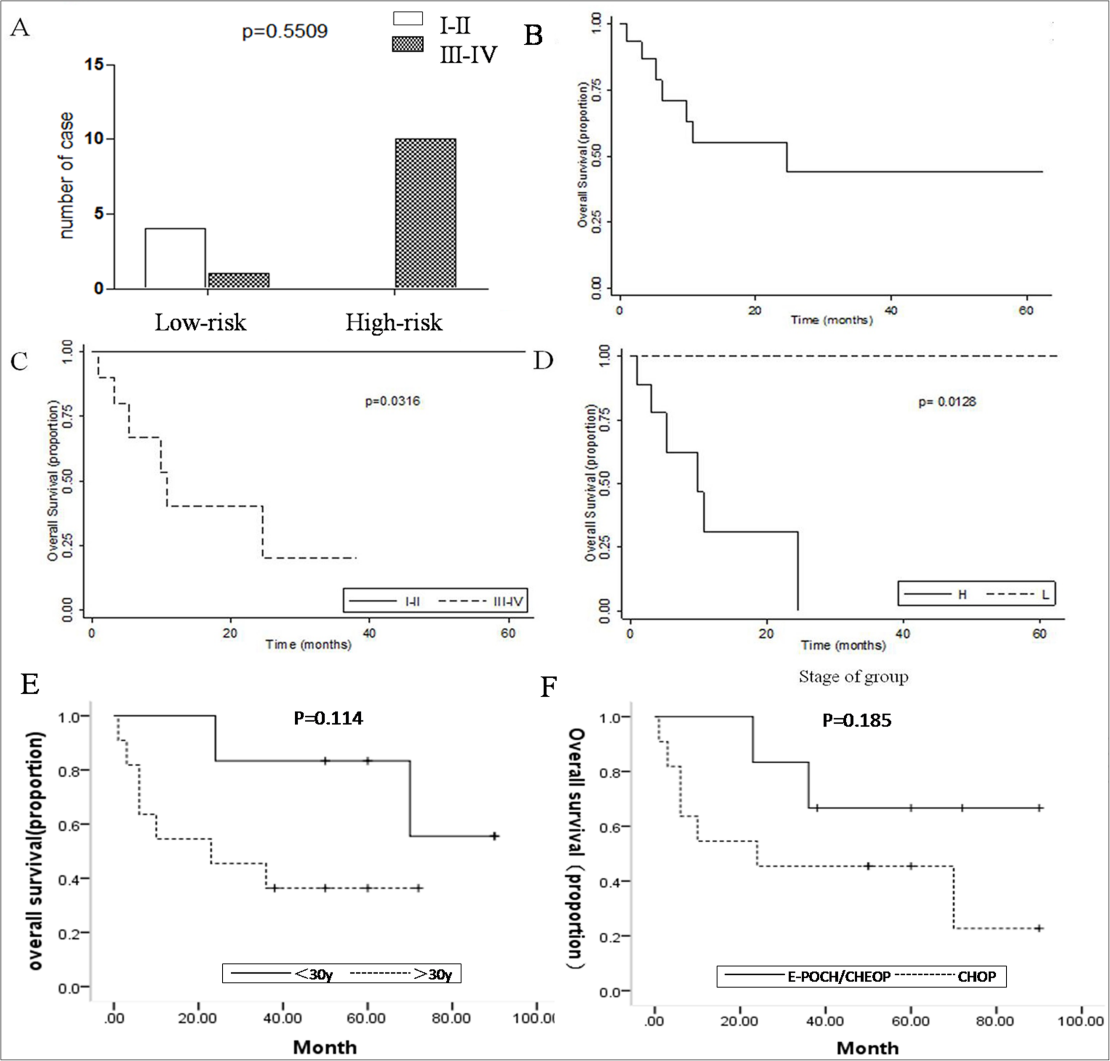
A: Distribution of both low- and high-risk groups according to the Ann Arbor staging criteria B: OS of all the cases C: OS of the low-risk and high-risk groups. D: Ann Arbor staging significantly impacted the OS. E: OS of the <30 y and >30 y groups. F: OS of the CHOP and CHOEP/E-POCH regimen groups.

Clinical information on the 17 cases was summarized in Table 1. These cases showed a remarkable male predominance with a ratio of 16:1. The average age was 39.6 years old and ranged from 12 to 72 years. Thirteen cases presented as lymphadenopathy, of which 10 cases were located in the cervical region; 5 cases presented as retroperitoneal lymphadenopathy accompanied by abdominal pain and inguinal lymph node enlargement. Extranodal occurrence was observed in 2 cases and involved the duodenum and tonsil. Bone marrow involvement was also observed in 4 cases. Nine (60%) patients presented with B symptoms. The serum lactate dehydrogenase (LDH) level was elevated in 15 documented patients, and HIV serology was negative. All patients but one underwent complete staging with clinical examination and radiologic studies. Among 16 documented patients, the majority experienced an aggressive clinical course; among these, cases of stage III-IV disease accounted for a large proportion (11/16) according to the Ann Arbor criteria. The International Prognostic Index (IPI) score was available for 15 patients. Based on the results of the IPI score, we divided the patients into two groups: the low-risk (score 0–2) group and the high-risk (score 3–5) group. The low-risk group contained 5 patients while the high-risk group contained 10. For the 5 low-risk cases, four were early stage (stage I-II) cases, and only one was in stage III-IV; all cases in the high-risk group were in an advanced stage (stage III, IV, *p* = 0.5509) (Fig 1A).

**Table 1:** Clinical information and follow up data of 17 patients.

Case	Gender/Age(y)	Involvement	Stage	Serum LDH	IPI score	Treatment	Survival(months)
1	M/23	mediastinal,retroperitoneal	IIIA	elevated	4	CHOP/E-POCH	10/DOD
2	M/72	Bilateral submandibular, cervical	IVA	elevated	5	CHOP/E-POCH	NA/NA
3	M/57	bones, bilateral cervical lymph node, mediastinal	IVB	elevated	4	CHOP	3/DOD
4	M/55	Bilateral cervical lymph node	IIA	normal	1	CHOP/E-POCH plus RT	48/Alive
5	M/25	Right cervical lymph node	IIA	411	2	CHOP plus RT	24/Alive
6	M/32	Right cervical lymph node	NA	207	NA	CHOP plus RT	6/DOD
7	M/68	Right cervical lymph node, retroperitoneal	IIIA	elevated	4	CHOP	10/DOD
8	M/33	Left supraclavicular,mediastinal, perigastric	IIIB	346	3	CHOP+MX+VP-16+BLM+DXM plus RT plus HYPER-CVAD-A	24/DOD
9	M/12	Cervical lymph node	IA	NA	NA	CHOP	6/Alive
10	M/22	Cervical lymph node, subraclavicular	IIIB	177	2	CHOP	27/Alive
11	M/26	Duodenum	IE	183	1	NA	NA/NA
12	M/42	Left tonsil, retroperitoneal	IVB	237	4	CEHOP	10/Alive
13	M/54	Left cervical	IIB	200	0	CHOEP plus RT plus HYPER-CVAD-A and HYPER-CVAD-B	8/Alive
14	M/20	Left groin, retroperitoneal, bone marrow	IVB	367	3	CHOP	6/Alive
15	M/52	Systemic lymph nodes	IVB	300	4	CHOP plus RT	6/DOD
16	F/47	Left groin, mediastinal	IVB	242	4	without regimens	1/DOD
17	M/34	Left cervical,bilateral hilar, retroperitoneal	IIIB	194	3	CHEOP	3/Alive

**Abbreviations:** LDH, lactate dehydrogenase; M, male; F, female; CHOP, cyclophosphosphamide, doxorubicin, vincristine, and prednisone; CHOEP/E-POCH, cyclophosphosphamide, doxorubicin, vincristine, and prednisone with etoposide or bleomycin; RT, radiotherapy; DOD, dead of disease; NA, not available.

### Treatment and outcome

Among the 16 documented patients, 10 were treated with CHOP or CHOP-like regimens for six cycles, and 5 were treated with an E-POCH/CHOEP regimen. Among them, 5 patients were treated with additional radiotherapy at as dose of 30 Gray. Two patients were treated with hyper-CVAD and multiple chemotherapy regimens after relapse. The duration of follow-up ranged from 1 to 90 months (mean time, 40.5 months). Information on the clinical outcomes was available for 15 patients. Most patients experienced an aggressive clinical course: 6 of them died within the first year, and one even died before treatment. For those who died of disease, the average survival and median survival were 8.6 and 8.0 months, respectively, and the 5-year OS was only 40% (Fig 1B). Patients with stages I-II disease had a significantly better 5-year OS (100%) than those with stages III-IV disease (5-y OS, 20%), as illustrated in (Fig 1C, *p* = 0.0128). Moreover, the OS of the low-risk group was also better compared with that of the high-risk group (Fig 1D) (*p* = 0.0316). Complete remission could be achieved in all patients who were categorized with early-stage disease (I-II), but only 55% of the patients with end-stage disease (III-IV) achieved complete remission. Among those who experienced complete remission, 2 patients experienced relapse and eventually succumbed to the disease, even if they were treated with hyper-CVAD and multiple chemotherapies. Seven patients who had died of the disease all belonged to the high-risk group and the stage III-IV group. Younger patients (under 30 y) had a significantly better OS than older patients (over 30 y) (*p* = 0.114) (Fig 1E). The Kaplan-Meier curve showed that the OS of the group that received the E-POCH/CHOEP regimen was better compared with that of the group that received the CHOP regimen (*p* = 0.185) (Fig 1F).

### Pathological features and Immunohistochemical findings

**Fig 2:**
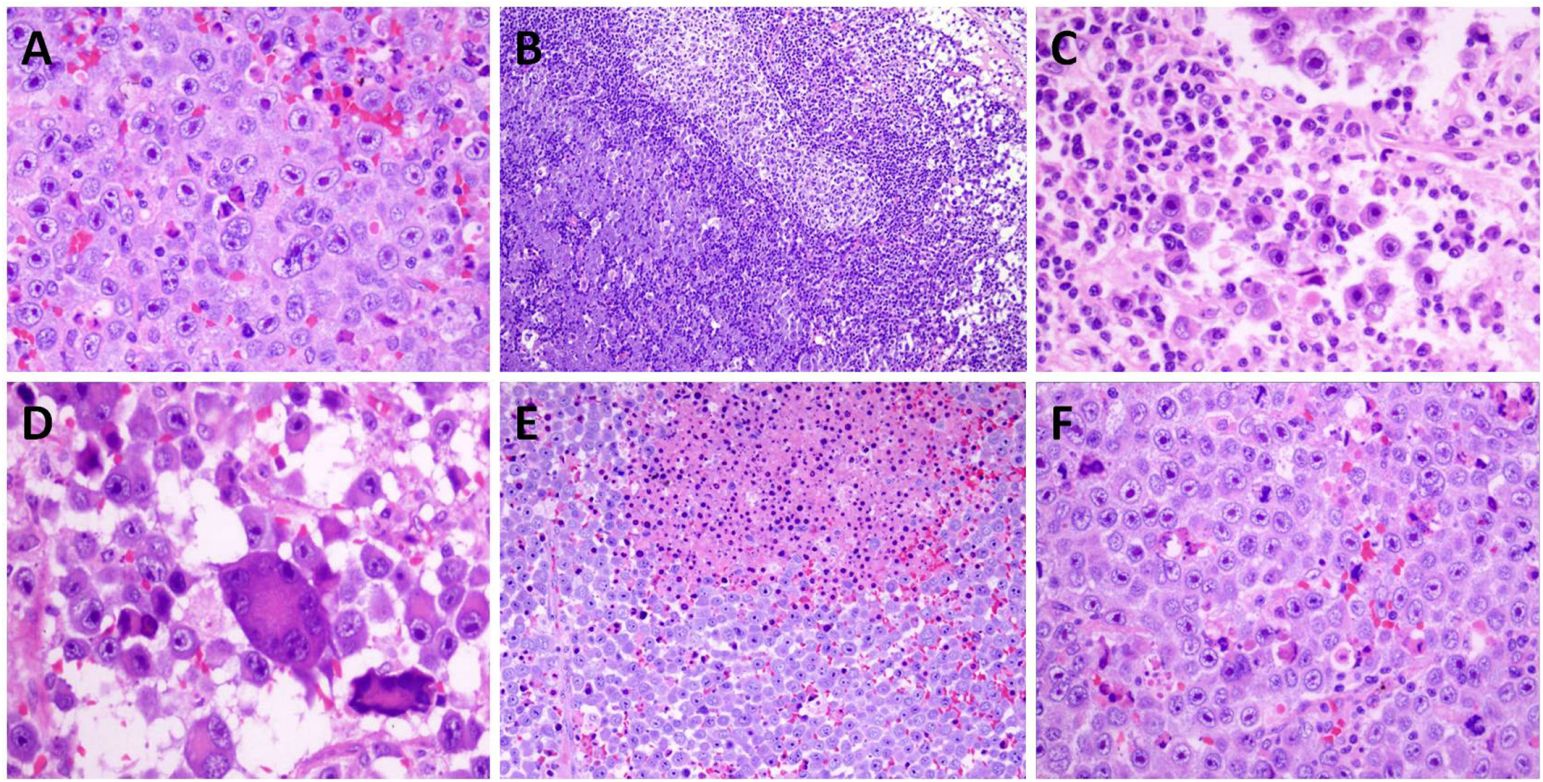
Histopathologic features of ALK-positive diffuse large B cell lymphoma. (haematoxylin & eosin). A: Medium- to large-sized cells with prominent nucleoli B: Sinusoidal Invasion C: plasmablastic/immunoblastic differentiation D: Multinucleated giant neoplastic cells E: Focal necrosis F: frequent mitosis.

**Fig 3:**
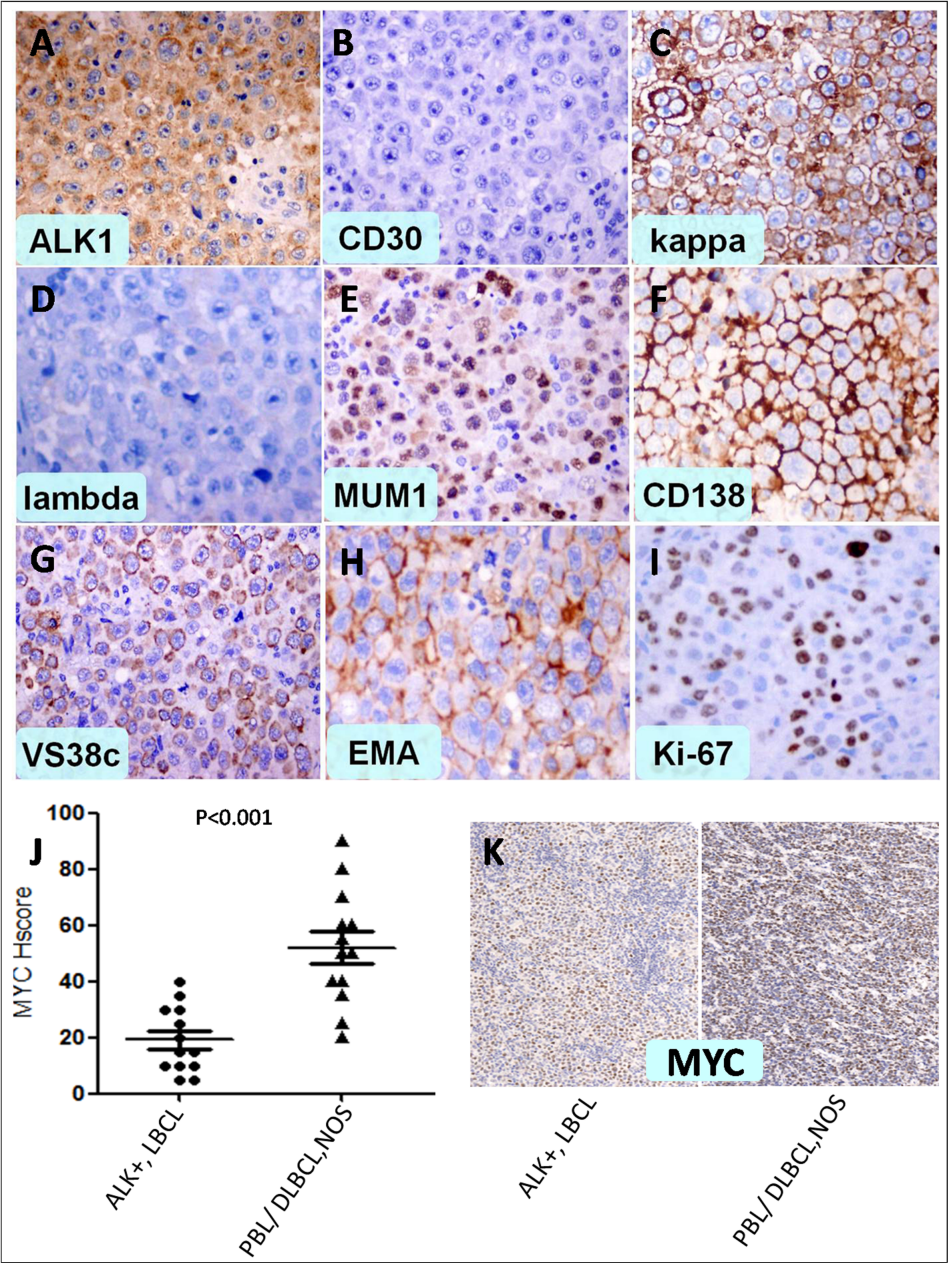
A: Monomorphic lymphoma cells expressing ALK with a cytoplasmic granular staining pattern B: Negativity for CD30 C, D: Kappa and Lambda show light chain restriction E, F, G, H: Expression of MUM1, CD138, VS38C and EMA I: Ki-67 staining shows a high proliferation index J, K: H-score were significant lower than that in Plasmablastic lymphoma (PBL) or diffuse large B-cell lymphoma, not otherwise specified (DLBCL, NOS).

Histologically, the tumours showed a diffuse infiltration of neoplastic cells in most cases. Sinusoidal invasion was observed in 5 cases, and this phenomenon was remarkable in cases that involved the lymph nodes. The neoplastic cells were uniformly medium- to large-sized with round nuclei; they also featured dispersed chromatin and single, central, prominent nucleoli. Typical, moderate amounts of eosinophilic to amphophilic cytoplasm were present, and the tumour cells presented with an extremely obvious plasmablastic/immunoblastic differentiation in almost every case. A large number of multinucleated giant neoplastic cells were obvious in 3 cases. This characteristic may frequently be found in older patients with high IPI scores and advanced stage disease. Focal necrosis was also observed in 3 cases. Small lymphocytes and plasma cells were present in varying proportions among the tumour cells (Fig 2). Details of the immunohistochemical findings are summarized in S2 Table. The tumour cells were positive for B-lineage markers including Oct-2 and Bob-1 as well as ALK in all cases, while a large proportion of cases were positive for plasma cell markers including CD38, CD138, MUM1 and VS38C (more than 75%). What’s more, C-MYC was also performed in 13 cases, the H-score were significant lower than that in Plasmablastic lymphoma (PBL) or diffuse large B-cell lymphoma, not otherwise specified (DLBCL, NOS) (*p*<0.001) (Fig 3).

### Molecular analysis

**Fig 4:**
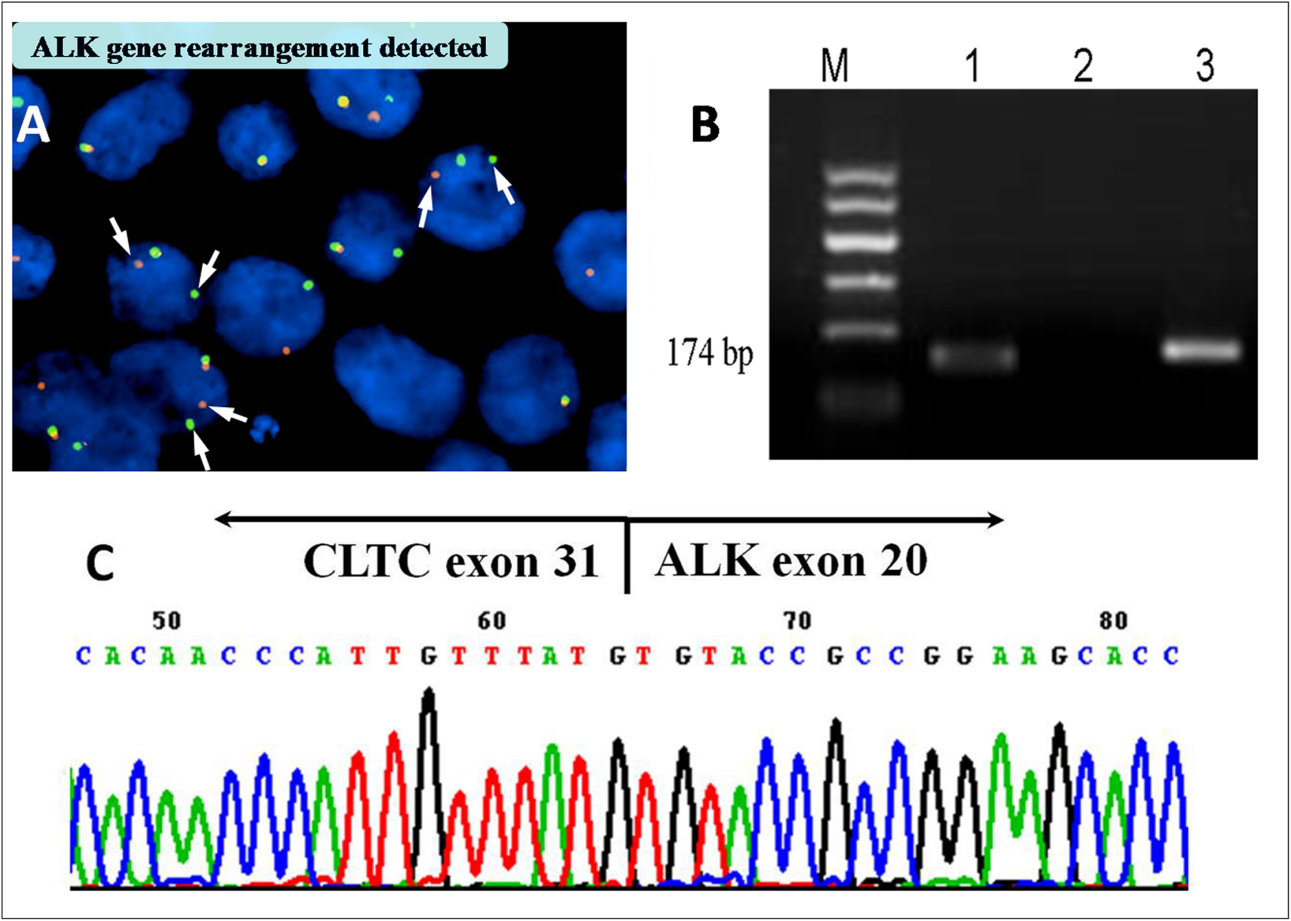
A: Positive FISH for the ALK break apart probe (white arrows). B: PCR electrophoretic band; M: Molecular weight marker, Lane 1: CLTC-ALK, Lane 2: Negative control, Lane 3: PGK. C: DNA sequencing suggests that the fusion partner is CLTC.

FISH was performed on 5 cases, and all of them showed a split of the dual-colour probe flanking *ALK*, which indicates a chromosomal break within the *ALK* gene (Fig 4A). Among these, chromosomal breaks were also detected by RT-PCR in four cases (Fig 4B), which suggests that the fusion partner was *CLTC*; this was further confirmed by DNA sequencing (Fig 4C). PCR analysis for *IGH* and *TCR* gene rearrangements was also performed, and 13 cases indicated monoclonal *IGH* rearrangement. No monoclonal *TCR* gene rearrangement was detected.

## Discussion

### Review of 151 cases

Approximately 134 cases of ALK+ LBCL have been reported in the literature [17–19]. The brief clinicopathologic features of the cases from the literature and those from our institution are all summarized in Table 2. ALK+ LBCL was observed predominantly in male patients, with a male to female ratio of 3.5:1. The average age of the patients was 38.4 years, and it ranged from 9 to 72 years. From an analysis of 106 patients whose records contained detailed information on age, only 18.9% (20/106) occurred in a paediatric population (18 y of age or younger). ALL cases in this study and those reported in the literature were positive for ALK protein. By EBER-ISH, EBV was not detected in any of the 87 cases. Detection of *IGH* and/or *IGK* gene rearrangement was performed in 43 cases, 34 (86%) of which showed positive B-cell monoclonality. Conventional cytogenetic and FISH assays detected *ALK* rearrangements in 94.7% (72/76) cases of ALK+ LBCL in our study and in the literature review [20,21]. Thirty-three cases had documented partner genes of *ALK* rearrangements, mostly *ALK-CLTC*, as noted in 75.7% of the cases (28/37); other partner genes included *NPM1* (4 cases), *SEC31A* (3 cases), *SQSTM1* (1 case), *RANBP2* (1 case), and IGL (1 case) [22–29].

**Table 2:** Summary of the clinicopathologic fueatures of the ALK+, LBCL cases of our study and the reviewed literatures.

	Our study	Literature	Total (%)
Male/female	16/1	99/28	115/29
Average age	39.6 (n = 17)	38.4 (n = 129)	39 (n = 146)
Primary sites(nodal/extranodal)	15/2	98/28	113/30
B symptoms	9/15	17/34	26/49 (53)
Bone marrow involvement	2/17	19/63 (30)	11/80 (13)
Clinical stage(I-II vs. III-IV)	4/11	43/58	47/69(68.1)
Average follow-up(mo)	40.5(n = 17)	23.8 (n = 92)	32.1 (n = 109)
Outcome(died/alive)	8/15	45/46	53/61
ALK	17/17	125/125	142/142 (100)
Bob.1	5/5	18/18	23/23 (100)
CD3	0/17	0/71	0/88 (0)
CD4	2/9	35/66	37/75 (49.3)
CD20	2/15	4/120	6/135 (4.4)
CD30	1/15	14/117	15/132 (11.4)
CD79a	3/9	21/108	24/117 (20.5)
CD138	9/11	101/107	110/118 (93.2)
IgA	0/2	60/75	60/77 (77.9)
Κ	5/9	25/61	30/70 (42.9)
λ	4/9	36/68	40/77 (51.9)
MUM1	8/9	45/54	53/63 (84.1)
Oct-2	7/7	20/23	27/30 (90)
EBER ISH	0/17	0/70	0/84 (0)
ALK rearrangement	4/5	67/71	71/76 (93.4)
*IGH* PCR	13/17	24/26	37/43 (86)

### Prognostic factors of ALK+ LBCL

ALK-positive LBCL is an aggressive subtype of diffuse large B cell lymphoma as more than 76% of the documented cases were in stage III-IV. In our study, we found that both the IPI score and the stage were significant prognostic factors [Fig 1C and Fig 1D]. The survival of patients in the high-risk group appeared to be worse than that of patients in the stage III-IV group, and the reason for this may be that a low IPI score case was categorized into the advanced stage group, which is what we can learn from Fig 1A. This case was considered stage III because of the wide range of involved lymph node regions. However, in terms of age, physical condition and other laboratory examination indices, this patient still belonged to the low-risk group. Additionally, younger patients (under 30 y) had a significantly better OS than older patients (over 30 y), but according to the *P*-value, the difference was not statistically significant. It was reported in the literature that patients younger than 35 years had a significantly better OS than those older than 35 years [19].

### Conventional therapies for ALK+, DLBCL

Our study was the second large cohort that suggested that ALK-positive LBCL lacks CD20 in all cases. Therefore, the efficacy of Rituximab is insufficient for the improvement of the outcome in this subset of cases. From a clinical perspective, ALK+, LBCL should be distinguished from typical DLBCL, which requires a distinct treatment. According to our review, most patients received chemotherapy, including CHOP, CHOEP, EPOCH, and CVAD, and some of them also underwent localized radiotherapy and haematopoietic stem cell transplantation. However, patients with ALK+ LBCL still displayed a dismal clinical outcome compared with patients with typical DLBCL who were treated with CHOP or CHOP-like regimens. Similarly, approximately 37.5% of cases at our institution received more rigorous chemotherapy such as E-CHOP, CHOEP, or E-POCH/CHOEP; these regimens do help to improve the survival compared with the CHOP regimen, but according to the *P*-value, the difference among regimens was not statistically significant [Fig 1F]. Additionally, both the analysis of our cases and a review of previously reported cases show that ALK + DLBCL can be an aggressive malignancy that can be cured with conventional chemotherapy protocols only at the stage of localized disease [19].

### Novel front-line therapy regimens

The highly aggressive nature of this lymphoma and the relative paucity of molecular data available highlight the need for deeper insights into the molecular pathogenesis of ALK-positive large B-cell lymphomas to identify new and effective alternative treatments. One research indicated that ALK-positive large B-cell lymphomas express a complete plasmablastic differentiation program but, contrary to plasmablastic lymphomas, do not have MYC rearrangements [30]. It was consistent with the low MYC expression level in our study, which suggested that MYC may not be the main molecular pathogenesis for the highly aggressive nature of ALK+, LBCL. Recent developments have led to significant diagnostic and therapeutic advances, including efficient diagnostic tests and ALK-targeting agents that are readily available in the clinical setting. Inhibition of ALK activity resulted in sustained tumour regression in a xenotransplant tumour model. These data indicate a role for CLTC-ALK in the maintenance of the malignant phenotype, which provides a rationale for a therapeutic target for these otherwise refractory tumours [31, 32]. To further study their therapeutic potential in ALK+, LBCL, a CTLC-ALK-positive LBCL cell line (LM1) was established as a preclinical model to study the role of CLTC- ALK activity in DLBCL lymphomagenesis. It was demonstrated that these lymphomas display activation of ALK signalling pathways and are potently suppressed in vitro and in vivo by a selective ALK inhibitor. The selective ALK inhibitor NVP-TAE684 repressed ALK-activated signalling pathways and induced apoptosis of LM1 DLBCL cells [33, 34]. Based on the molecular pathology described above, the recent introduction of the small molecule ALK inhibitor crizotinib may provide a potential new therapeutic option for patients with this disease.

Unquestionably, research on different therapies has been very productive in recent years and will most likely continue to be in the future. Another recent study suggests that ALK expression in DLBCL is strictly linked to STAT3 phosphorylation. STAT3 would be one of the molecular targets in ALK-positive DLBCL. The relevance of the ALK/STAT3 pathway in the pathogenesis of ALK-positive large B-cell lymphomas indicates that this pathway is an attractive target for new therapies [35, 36]. Specifically, STAT3 inhibition also serves as a possible therapeutic target for lymphomas with the SQSTM1-ALK variant translocation [37].

## Conclusions

ALK-positive large B cell lymphoma is a rare tumour with characteristic pathological and clinical feature. Morphologic analysis, immunohistochemistry and genetic tests show that the *ALK* gene is associated as an external cause of the disease but that the disease is part of a spectrum. ALK +, LBCL is defined as a separate entity but may belong to this broad spectrum. The recognition of ALK+, LBCL as a distinct entity is important because most patients experience an aggressive disease course and are candidates for novel treatment approaches. Further prospective studies are needed to optimize therapies for this disease.

## Supporting information

S1 Table Primers for the detection of ALK fusion transcripts. (DOCX)

S2 Table Summary of immunohistochemical findings. (DOCX)

S1 Data URLS and DOIs of S1 Table and S2 Table. (DOC)

## References

[1] Delsol G., Lamant L., Mariam´e B., Pulford K., Dastugue N., Brousset P. et al A new subtype of large B-cell lymphoma expressing the ALK kinase and lacking the 2;5 translocation. Blood. 1997; 89:1483–1490. 9057627

[2] S. Swerdlow, E. Campo, N. L. Harris, E. S. Jaffe, S. A. Pileri, and H. Stein,WHO Classification of tumors of hematopoietic and lymphoid tissues, 2008.

[3] Takeuchi K, Soda M, Togashi Y, Ota Y, Sekiguchi Y, Hatano S et al Identification of a novel fusion, SQSTM1-ALK, in ALK-positive large B-cell lymphoma. Haematologica. 2011;96:464–7. doi: 10.3324/haematol.2010.033514 21134980 10.3324/haematol.2010.033514 PMC3046280

[4] Laurent C, Do C, Gascoyne RD, Lamant L, Ysebaert L, Laurent G et al Anaplastic lymphoma kinase-positive diffuse large B-cell lymphoma: a rare clinicopathologic entity with poor prognosis. J Clin Oncol. 2009;27:4211–6. doi: 10.1200/JCO.2008.21.5020 19636007 10.1200/JCO.2008.21.5020

[5] Gascoyne RD, Lamant L, Martin-Subero JI, Lestou VS, Harris NL, Müller-Hermelink HK et al ALK-positive diffuse large B-cell lymphoma is associated with Clathrin-ALK rearrangements: report of 6 cases. Blood. 2003;102:2568–73. doi: 10.1182/blood-2003-03-0786 12763927 10.1182/blood-2003-03-0786

[6] Rudzki Z, Rucinska M, Jurczak W, Skotnicki AB, Maramorosz-Kurianowicz M, Mruk A et al ALK-positive diffuse large B-cell lymphoma: two more cases and a brief literature review. Pol J Pathol. 2005;56:37–45. 15921012

[7] Wang WY, Ma ZG, Li GD, Liu WP, Zhong L, Wang Y, et al Diffuse large B-cell lymphoma with expression of anaplastic lymphoma kinase protein: clinicopathologic and immunohistochemical study of 5 cases. Zhonghua Bing Li Xue Za Zhi. 2006;35:529–34. 17134546

[8] Lee HW, Kim K, Kim W, Ko YH. ALK-positive diffuse large B-cell lymphoma: report of three cases. Hematol Oncol. 2008;26:108–13. doi: 10.1002/hon.841 18220322 10.1002/hon.841

[9] Beltran B, Castillo J, Salas R, Quiñones P, Morales D, Hurtado F, et al ALK-positive diffuse large B-cell lymphoma: report of four cases and review of the literature. J Hematol Oncol. 2009;2:11 doi: 10.1186/1756-8722-2-11 19250532 10.1186/1756-8722-2-11 PMC2651189

[10] Li K, Tipps AM, Wang HY. Anaplastic lymphoma kinase-positive diffuse large B-cell lymphoma presenting as an isolated nasopharyngeal mass: a case report and review of literature. Int J Clin Exp Pathol. 2011;4:190–6. 21326807 PMC3037206

[11] Yu H, Huang JX, Wang CF, Shi DR. et al ALK-positive large B-cell lymphoma: report of a case. Zhonghua Bing Li Xue Za Zhi. 2011;40:561–2. 22169650

[12] Li K, Tipps AM, Wang HY. Anaplastic lymphoma kinase-positive diffuse large B-cell lymphoma presenting as an isolated nasopharyngeal mass: a case report and review of literature. Int J Clin Exp Pathol. 2011;4:190–6. 21326807 PMC3037206

[13] Chen YP, Hung LY, Shan YS, Chang KC. ALK-positive large B-cell lymphoma presenting with jejunal intussusception. Eur J Haematol. 2013;90:261 doi: 10.1111/ejh.12031 23171433 10.1111/ejh.12031

[14] Wass M, Behlendorf T, Schadlich B, Mottok A, Rosenwald A, Schmoll HJ, et al Crizotinib in refractory ALK-positive diffuse large B-cell lymphoma: a case report with a short-term response. Eur J Haematol. 2014;92:268–70. doi: 10.1111/ejh.12240 24330038 10.1111/ejh.12240

[15] Morgan EA, Nascimento AF. Anaplastic lymphoma kinase-positive large B-cell lymphoma: an underrecognized aggressive lymphoma. Adv Hematol. 2012;2012:529572 doi: 10.1155/2012/529572 22474449 10.1155/2012/529572 PMC3299366

[16] Vysis ALK break apart FISH probe kit protocol. [2013-03-30]. http://www.abbottmolecular.com/static/cms_workplace/pdfs/US/Vysis_ALK_FISH_Probe_Kit_PI.Pdf

[17] Gascoyne RD, Lamant L, Martin-Subero JI, Lestou VS, Harris NL, Müller-Hermelink HK, er al. ALK-positive diffuse large B-cell lymphoma is associated with Clathrin-ALK rearrangements: report of 6 cases. Blood. 2003;102:2568–2573 doi: 10.1182/blood-2003-03-0786 12763927 10.1182/blood-2003-03-0786

[18] Laurent C, Do C, Gascoyne RD, Lamant L, Ysebaert L, Laurent G, et al Anaplastic lymphoma kinase-positive diffuse large B-cell lymphoma: a rare clinicopathologic entity with poor prognosis. J Clin Oncol. 2009;27:4211–4216. doi: 10.1200/JCO.2008.21.5020 19636007 10.1200/JCO.2008.21.5020

[19] Pan Z, Hu S, Li M, Zhou Y, Kim YS, Reddy V, et al ALK-positive large B-cell lymphoma: A clinicopathologic study of 26 cases with review of additional 108 cases in the literature. Am J Surg Pathol. 2017 2;41(1):25–38. doi: 10.1097/PAS.0000000000000753 27740969 10.1097/PAS.0000000000000753

[20] Adam P, Katzenberger T, Seeberger H, Gattenlöhner S, Wolf J, Steinlein C, et al A case of a diffuse large B-cell lymphoma of plasmablastic type associated with the t(2;5)(p23;q35) chromosome translocation. Am J Surg Pathol. 2003;27:1473–6. 14576483 10.1097/00000478-200311000-00012

[21] De Paepe P, Baens M, van Krieken H, Verhasselt B, Stul M, Simons A, et al ALK activation by the CLTC-ALK fusion is a recurrent event in large B-cell lymphoma. Blood. 2003;102:2638–41. doi: 10.1182/blood-2003-04-1050 12750159 10.1182/blood-2003-04-1050

[22] Van Roosbroeck K, Cools J, Dierickx D, Thomas J, Vandenberghe P, Stul M, et al ALK-positive large B-cell lymphomas with cryptic SEC31A-ALK and NPM1-ALK fusions. Haematologica. 2010;95:509–513. doi: 10.3324/haematol.2009.014761 20207848 10.3324/haematol.2009.014761 PMC2833084

[23] Adam P, Katzenberger T, Seeberger H, Gattenlöhner S, Wolf J, Steinlein C, et al A case of a diffuse large B-cell lymphoma of plasmablastic type associated with the t(2;5)(p23;q35) chromosome translocation. Am J Surg Pathol. 2003;27:1473–1476. 14576483 10.1097/00000478-200311000-00012

[24] Onciu M, Behm FG, Downing JR, Shurtleff SA, Raimondi SC, Ma Z, et al ALK-positive plasmablastic B-cell lymphoma with expression of the NPM-ALK fusion transcript: report of 2 cases. Blood. 2003;102:2642–2644. doi: 10.1182/blood-2003-04-1095 12816858 10.1182/blood-2003-04-1095

[25] Bedwell C, Rowe D, Moulton D, et al Cytogenetically complex SEC31A-ALK fusions are recurrent in ALK-positive large B-cell lymphomas. Haematologica. 2011;96:343–346. doi: 10.3324/haematol.2010.031484 21109691 10.3324/haematol.2010.031484 PMC3031708

[26] Van Roosbroeck K, Cools J, Dierickx D, Jones G, Bown N, Bacon CM.et al ALK-positive large B-cell lymphomas with cryptic SEC31A-ALK and NPM1-ALK fusions. Haematologica. 2010;95:509–513. doi: 10.3324/haematol.2009.014761 20207848 10.3324/haematol.2009.014761 PMC2833084

[27] d’Amore ES, Visco C, Menin A, Famengo B, Bonvini P, Lazzari E.et al STAT3 pathway is activated in ALK-positive large B-cell lymphoma carrying SQSTM1-ALK rearrangement and provides a possible therapeutic target. Am J Surg Pathol. 2013;37:780–786. doi: 10.1097/PAS.0b013e318287791f 23588372 10.1097/PAS.0b013e318287791f

[28] Stachurski D, Miron PM, Al-Homsi S, Hutchinson L, Harris NL, Woda B, et al Anaplastic lymphoma kinase-positive diffuse large B-cell lymphoma with a complex karyotype and cryptic 3' ALK gene insertion to chromosome 4 q22-24. Hum Pathol. 2007;38:940–5. doi: 10.1016/j.humpath.2006.12.019 17509395 10.1016/j.humpath.2006.12.019

[29] Shi M, Miron PM, Hutchinson L, Woda BA, Nath R, Cerny J, et al Anaplastic lymphoma kinase-positive large B-cell lymphoma with complex karyotype and novel ALK gene rearrangements. Hum Pathol. 2011;42:1562–7. doi: 10.1016/j.humpath.2011.01.012 21497367 10.1016/j.humpath.2011.01.012

[30] Valera A, Colomo L, Martinez A, de Jong D, Balague O, Matheu G, et al ALK-positive large B-cell lymphomas express a terminal B-cell differentiation program and activated STAT3 but lack MYC rearrangements. Mod Pathol 2013;26:1329–37. doi: 10.1038/modpathol.2013.73 23599149 10.1038/modpathol.2013.73 PMC6368829

[31] Cerchietti L, Damm-Welk C, Vater I, Klapper W, Harder L, Pott C, et al Inhibition of anaplastic lymphoma kinase (ALK) activity provides a therapeutic approach for CLTC-ALK-positive human diffuse large B cell lymphomas. PLoS One. 2011;6:e18436 doi: 10.1371/journal.pone.0018436 21494621 10.1371/journal.pone.0018436 PMC3072987

[32] Wass M, Behlendorf T, Schadlich B, Mottok A, Rosenwald A, Schmoll HJ, et al Crizotinib in refractory ALK-positive diffuse large B-cell lymphoma: a case report with a short-term response. Eur J Haematol. 2014;92:268–70. doi: 10.1111/ejh.12240 24330038 10.1111/ejh.12240

[33] Galkin AV, Melnick JS, Kim S, Hood TL, Li N, Li L, et al Steensma R, Chopiuk G, Jiang J and others. Identification of NVP-TAE684, a potent, selective, and efficacious inhibitor of NPM-ALK. Proc Natl Acad Sci U S A. 2007;104:270–5. doi: 10.1073/pnas.0609412103 17185414 10.1073/pnas.0609412103 PMC1765448

[34] Cheng M, Quail MR, Gingrich DE, Ott GR, Lu L, Wan W, et al CEP-28122, a highly potent and selective orally active inhibitor of anaplastic lymphoma kinase with antitumor activity in experimental models of human cancers. Mol Cancer Ther. 2012;11:670–9. doi: 10.1158/1535-7163.MCT-11-0776 22203728 10.1158/1535-7163.MCT-11-0776

[35] Valera A, Colomo L, Martinez A, de Jong D, Balagué O, Matheu G, et al ALK-positive large B-cell lymphomas express a terminal B-cell differentiation program and activated STAT3 but lack MYC rearrangements. Mod Pathol. 2013;26:1329–37. doi: 10.1038/modpathol.2013.73 23599149 10.1038/modpathol.2013.73 PMC6368829

[36] Momose S, Tamaru J, Kishi H, Mikata I, Mori M, Toyozumi Y, et al Hyperactivated STAT3 in ALK-positive diffuse large B-cell lymphoma with clathrin-ALK fusion. Hum Pathol. 2009;40:75–82. doi: 10.1016/j.humpath.2008.06.009 18755494 10.1016/j.humpath.2008.06.009

[37] D'Amore ES, Visco C, Menin A, Famengo B, Bonvini P, Lazzari E. et al STAT3 pathway is activated in ALK-positive large B-cell lymphoma carrying SQSTM1-ALK rearrangement and provides a possible therapeutic target. Am J Surg Pathol. 2013;37:780–6. doi: 10.1097/PAS.0b013e318287791f 23588372 10.1097/PAS.0b013e318287791f

