# Supplementary material for: Anaplastic lymphoma kinase-positive large B-cell lymphoma: Clinico-pathological study of 17 cases with review of literature
Source: PLoS One. 2017 Jun 30;12(6):e0178416. doi: 10.1371/journal.pone.0178416 (PMC5493294; doi:10.1371/journal.pone.0178416)
Supplement: S1 Table — (DOCX) [file pone.0178416.s001.docx]

| **Primer** | **Sequence** | **PCR product Size(bp)** |
| --- | --- | --- |
| TPM3/4-f | 5’ –AGACAATTGATGACCTGGAAGTG -3’ | 100 |
| CLTC-f | 5’ –GAAGGAGTACTTGACAAAGGTGGAT -3’ | 174 |
| CARS-f | 5’ –CGAGAAGGAGTGCGGAAGAT -3’ | 116 |
| NPM-f | 5’ –AAGTGTGGTTCAGGGCCAGT -3’ | 125 |
| TFG-f | 5’ –CAGCAGCCACCATATACAGGA -3’ | 128 |
| ATIC-f | 5’ –TGGAATGAACCCACATCAGAC -3’ | 144 |
| ALO17-f | 5’ –TGAAGATGTGTGGGAACGTG -3’ | 115 |
| ALK-r | 5’ –CGGAGCTTGCTCAGCTTGTA -3’ |  |

**【Table1】 Primers for the detection of ALK fusion transcripts**
