# Supplementary material for: Anaplastic lymphoma kinase-positive large B-cell lymphoma: Clinico-pathological study of 17 cases with review of literature
Source: PLoS One. 2017 Jun 30;12(6):e0178416. doi: 10.1371/journal.pone.0178416 (PMC5493294; doi:10.1371/journal.pone.0178416)
Supplement: S2 Table — (DOCX) [file pone.0178416.s002.docx]

| **Marker** | **No. of patients with positive results** | **No. of patients tested** | **(%)** |
| --- | --- | --- | --- |
| CD45RB | 4 | 7 | 57.14 |
| CD20 | 2 | 15 | 13.33 |
| Oct-2 | 7 | 7 | 100 |
| Bob-1 | 5 | 5 | 100 |
| MUM1 | 8 | 9 | 88.89 |
| kappa | 5 | 9 | 55.56 |
| lambda | 4 | 9 | 44.45 |
| AE1/AE3 | 2 | 8 | 25 |
| CD79a | 3 | 9 | 33.33 |
| Ki-67 | 17(60%-90%) | 17 | 100 |
| IgA | 0 | 2 | 0 |
| CD38 | 3 | 9 | 33.33 |
| CD138 | 9 | 11 | 81.81 |
| VS38C | 4 | 4 | 100 |
| CD3 | 0 | 12 | 0 |
| CD4 | 2 | 9 | 22.22 |
| CD57 | 5 | 9 | 55.56 |
| CD30 | 1 | 15 | 6.67 |
| ALK | 17 | 17 | 100 |
| EMA | 11 | 14 | 78.57 |
| CD30 | 0 | 17 | 0 |
| MYC | 10(10%-55%) | 13 | 76.9 |

**【Table3】summary of immunohistochemical findings**
