# Supplementary material for: Anaplastic lymphoma kinase-positive large B-cell lymphoma: Clinico-pathological study of 17 cases with review of literature
Source: PLoS One. 2017 Jun 30;12(6):e0178416. doi: 10.1371/journal.pone.0178416 (PMC5493294; doi:10.1371/journal.pone.0178416)
Supplement: S1 Data — (DOC) [file pone.0178416.s003.doc]

URLs:<https://figshare.com/articles/s1_docx_s2_dox_Xiang-Nan_Jiang/5047501>

Jiang, Xiang-Nan; Yu, Bao-Hua; Wang, Wei-Ge; Zhou, Xiao-Yan; Li, Xiao-Qiu (2017): s1.docx, s2.dox Xiang-Nan Jiang. figshare.

DOIs: [**https://doi.org/10.6084/m9.figshare.5047501.v1**](https://doi.org/10.6084/m9.figshare.5047501.v1)

Retrieved: 13 07, May 27, 2017 (GMT)

Jiang, Xiang-Nan; Yu, Bao-Hua; Wang, Wei-Ge; Zhou, Xiao-Yan; Li, Xiao-Qiu (2017): s1.docx, s2.dox Xiang-Nan Jiang. figshare.

Retrieved: 13 07, May 27, 2017 (GMT)
